# A novel antiviral role of ankyrin repeat and LEM domain-containing 2 (ANKLE2) in restricting vaccinia virus through barrier to autointegration factor (BAF)

**DOI:** 10.1128/jvi.01549-25

**Published:** 2025-11-18

**Authors:** Kepalee Saeng-chuto, Zhigang Wang, Alexandria C. Krueger, Kaylee Bargeron, Matthew S. Wiebe

**Affiliations:** 1 School of Veterinary Medicine and Biomedical Sciences, University of Nebraska 684783, Lincoln, Nebraska, USA; 2 Nebraska Center for Virology, University of Nebraska 14719, Lincoln, Nebraska, USA; 3 School of Biological Sciences, University of Nebraska 118735, Lincoln, Nebraska, USA; Northwestern University Feinberg School of Medicine, Chicago, Illinois, USA

**Keywords:** ANKLE2, BAF, B1, phosphorylation, antiviral activity, poxvirus, vaccinia virus

## Abstract

ANKLE2 (Ankyrin repeat and LEM domain–containing protein 2) is an emerging host factor with previously undefined roles in antiviral defense. Here, we show that ANKLE2 can exert robust antiviral activity against vaccinia virus by regulating the phosphorylation status of barrier-to-autointegration factor (BAF), a ubiquitous DNA-binding protein that compacts DNA and restricts viral replication. We first demonstrate that depletion of endogenous ANKLE2 increased BAF phosphorylation and rescued replication of B1-knockout vaccinia virus, whereas reconstitution restored restriction. We then perform domain-mapping experiments of ANKLE2, revealing that its LEM domain and Caulimovirus domain (CD domain) are essential for BAF dephosphorylation, ANKLE2-BAF association, and/or antipoxviral activity, whereas the transmembrane (TM) domain restricts cytoplasmic redistribution and functions as a negative regulator. Together, these findings uncover a previously unrecognized host defense pathway against poxviruses, provide new insight into how host ANKLE2 proteins coordinate antiviral responses, and reveal a novel antiviral role for ANKLE2 in limiting vaccinia virus DNA replication and progeny release through regulation of BAF phosphorylation.

**IMPORTANCE:**

Vaccinia virus relies on disabling host defenses to replicate efficiently, with the host DNA-binding protein BAF representing a key target for viral kinases. Here, we uncover ANKLE2 as a critical host factor that counteracts vaccinia virus by sustaining the antiviral activity of BAF. ANKLE2 promotes BAF dephosphorylation, thereby preventing viral escape from BAF-mediated restriction. Our results reveal that distinct domains of ANKLE2 differentially regulate its antiviral activity, with the LEM and CD domains promoting BAF dephosphorylation and antiviral activity, and the transmembrane domain acting as a negative regulator by limiting cytoplasmic redistribution. These findings highlight ANKLE2 as a domain-dependent regulator of host defense and expand our understanding of the molecular circuitry that controls poxvirus replication.

## INTRODUCTION

Poxviruses are large double-stranded DNA (dsDNA) viruses that complete all stages of their life cycle in the cytoplasm of infected cells. Vaccinia virus, the prototypical member of the poxvirus family, has a 195-kilobase genome encoding approximately 200 proteins (1). Some proteins expressed during the early phase of the viral lifecycle are essential for initiating viral DNA replication and modulating host cell signaling to establish an environment favorable for viral survival and evasion of host immunity (2). During early infection, the vaccinia virus B1 protein, a well-characterized serine/threonine kinase, is produced and facilitates multiple stages of the virus life cycle through modulation of cellular signal transduction (3–12). During genome replication, B1 kinase promotes the vaccinia lifecycle by mimicking the activity of host vaccinia-related kinases (VRKs) to phosphorylate and inactivate the cellular antiviral activity of barrier-to-autointegration factor (BAF; encoded by the *BANF1* gene) (3, 8, 9, 13–15).

BAF is a small (∼10 kDa) highly conserved metazoan protein that not only acts as an antiviral effector against poxviruses but is also critical for maintaining cellular genome integrity in uninfected cells. In particular, BAF is known to contribute to nuclear reorganization and supports nuclear reassembly and nuclear envelope repair by acting as a molecular bridge between chromatin and inner nuclear membrane (INM) proteins containing LEM (LAP2-Emerin-MAN1) domains, which use this domain to interact directly with BAF (16–22). The DNA-binding activity of BAF is controlled through phosphorylation and dephosphorylation. Unphosphorylated BAF forms dimers that bind two strands of dsDNA in a sequence-independent manner, crossbridging and compacting the DNA, which underlies its effectiveness as an antiviral effector against vaccinia virus in the cytoplasm and is also a key property during cell division (3, 8, 23–28). BAF phosphorylation by the viral B1 kinase or cellular VRK1 kinase disrupts its DNA-binding capacity, thereby allowing efficient vaccinia virus DNA replication (8, 9, 13, 14, 29–31) or allowing for the regulation of key mitotic events in uninfected cells.

ANKLE2 (Ankyrin repeat and LEM domain-containing 2), also known as Lem4 or MCPH16, is a ubiquitously expressed gene (32), encoding a scaffolding protein belonging to the LEM family of INM proteins. ANKLE2 is comprised of several conserved domains, including a transmembrane (TM) domain, a LEM domain, a Caulimovirus domain (CD domain), an ankyrin-repeat (ANK) domain, and other purportedly structured regions of unknown function (33, 34). ANKLE2 localizes to the cytoplasmic face of the endoplasmic reticulum (ER) and the INM, where it interacts with kinases, phosphatases, and their substrates, and plays an important role in nuclear envelope reassembly during mitosis in signaling pathways shared by VRK1 (35, 36). Importantly, ANKLE2 is linked to multiple disease processes, including microcephaly (37, 38). Furthermore, a recent study provided the first evidence of a proviral activity of ANKLE2, showing clearly that it promotes Zika virus (ZIKV) replication by inducing membrane rearrangements that facilitate viral genome replication and shield viral double-stranded RNA (dsRNA) from immune detection (39). These roles in supporting a virus and in pathways governed by VRK1 partly informed our decision to investigate whether ANKLE2 also influences the replication of vaccinia virus.

Earlier studies suggest that during mitosis, ANKLE2 functions as a regulatory scaffold for serine/threonine protein phosphatase 2A (PP2A) to promote BAF dephosphorylation, with ANKLE2 knockdown resulting in hyperphosphorylation of BAF in *Caenorhabditis elegans, Drosophila,* and human HeLa cells (35, 40, 41). Interestingly, our previous studies indicated that PP2A contributes to BAF regulation during vaccinia infection. Specifically, depletion of PP2A caused accumulation of hyperphosphorylated BAF in cells, although this was not sufficient to enhance replication of a B1-deficient mutant vaccinia virus (42), which suggested that other regulators of PP2A may be at work as well. These findings encouraged us to investigate whether ANKLE2, as a binding partner of PP2A, has a more direct role in facilitating BAF activation, thereby acting as an antiviral effector against vaccinia virus replication using a similar pathway employed during mitosis. Herein, we investigate the molecular mechanism of ANKLE2-mediated inhibition of vaccinia, characterizing the impact of the TM, LEM, and CD domains of ANKLE2 in the activation of BAF antiviral activity via dephosphorylation.

## RESULTS

### Depletion of endogenous ANKLE2 disrupts BAF dephosphorylation and promotes ΔB1 vaccinia virus replication

**Fig 1:**
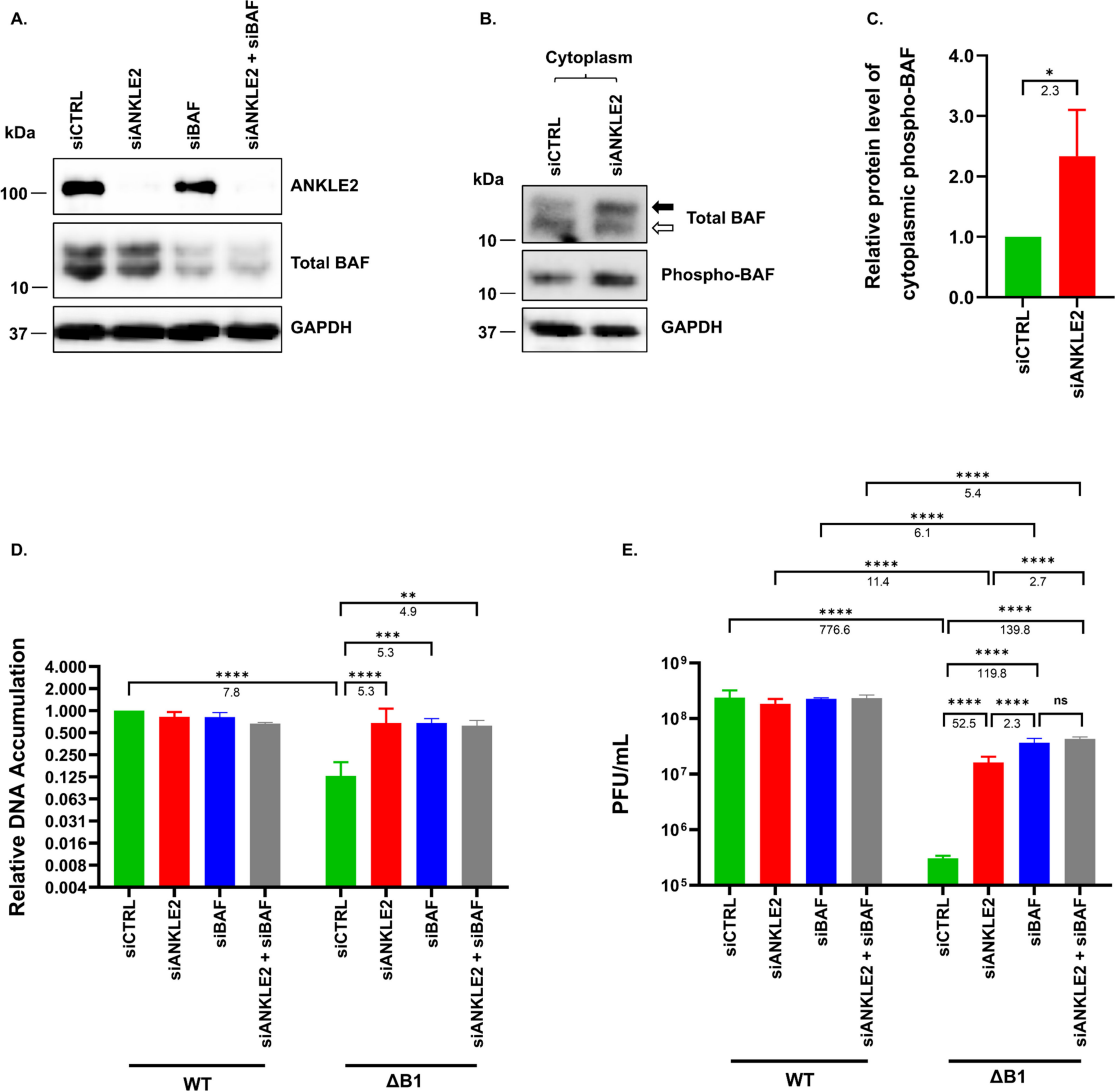
Impact of ANKLE2 depletion on BAF phosphorylation and WT or ΔB1 vaccinia virus infection in HeLa cells. (**A**) HeLa cells were transfected with siCTRL or siANKLE2 for 3 days, followed by immunoblotting analysis of ANKLE2. An immunoblot of GAPDH was included as a loading control. (**B**) Cytoplasmic BAF from siANKLE2-transfected HeLa cells was obtained by subcellular fractionation and analyzed by immunoblotting. Closed arrow indicates phosphorylated BAF. The open arrow indicates unphosphorylated BAF. An immunoblot of GAPDH was included as a loading control. (**C**) Relative phospho-BAF protein levels were quantified using ImageLab software (Bio-Rad) from the four independent replicates. The values for siCTRL-transfected cells were normalized to 1. (**D**) DNA accumulation and (**E**) viral yield were measured for WT or ΔB1 vaccinia virus at 24 hpi in siRNA-transfected HeLa cells infected at an MOI of 3. Data are from more than three independent experimental replicates. *, *P*  <  0.05; ***, *P*  <  0.001; ****, *P*  <  0.0001. Relative fold differences are given under each bracket.

BAF activity is controlled by dynamic phosphorylation, which modulates its binding to dsDNA, thus regulating its functions during mitosis and antiviral capability. Studies of the kinases involved in the regulation of BAF antiviral function have proceeded at a more rapid pace than the investigation of BAF dephosphorylation. To address this knowledge gap, we investigated the potential role of ANKLE2. ANKLE2 has an established role in regulating BAF phosphorylation during mitosis, but has not been studied during poxvirus infection. In this study, we investigated whether ANKLE2 also modulates vaccinia virus replication and whether this correlates with altered BAF phosphorylation. To test this, HeLa cells were transfected with a previously validated siRNA targeting ANKLE2 (siANKLE2) (35) and/or a siRNA targeting BAF (siBAF). Western blot analysis confirmed efficient knockdown of ANKLE2 and partial reduction of BAF compared to cells treated with control siRNA (siCTRL) (Fig. 1A). GAPDH levels were comparable across all samples, demonstrating that ANKLE2 depletion did not affect cell number or overall protein loading. We then performed subcellular fractionation followed by immunoblotting to determine whether phosphorylation of cytoplasmic BAF was impacted by loss of ANKLE2. Upon ANKLE2 depletion, a significant increase in phosphorylated cytoplasmic BAF levels was observed (2.3-fold) along with a decrease in unphosphorylated BAF (Fig. 1B and C), consistent with prior data indicating ANKLE2 facilitates BAF dephosphorylation.

We next inquired whether single or co-depletion of ANKLE2 and BAF impacted replication of vaccinia virus through the use of both wild-type (WT) virus and a viral knockout mutant lacking expression of the viral B1 kinase (ΔB1). This mutant was chosen because B1 is known to phosphorylate and inactivate BAF during WT virus infection, whereas growth of the ΔB1 virus is sensitive to BAF regulation by cellular enzymes (8, 9, 13, 30). For this study, HeLa cells were infected with either WT or ΔB1 vaccinia virus at an MOI of 3. At 24 h postinfection (hpi), neither single nor co-depletion of ANKLE2 and BAF had an effect on WT vaccinia virus DNA accumulation or production of viral progeny (Fig. 1D and E). In contrast, a significant rescue of ΔB1 vaccinia virus DNA accumulation was observed (Fig. 1D). This rescue effect extended to progeny production, as the ΔB1 vaccinia virus titer was significantly elevated following ANKLE2 (52.5-fold), BAF (119.8-fold), or co-depletion (139.8-fold) (Fig. 1E). Notably, co-depletion resulted in a significant increase in viral titer relative to ANKLE2 depletion alone (2.7-fold) but did not further elevate the titer beyond that observed with BAF depletion alone. Together, these findings affirm that ANKLE2 is involved in the regulation of BAF phosphorylation and demonstrate for the first time that ANKLE2 depletion rescues ΔB1 vaccinia virus replication. These correlating observations suggest that ANKLE2 may be needed to activate the antipoxviral function of BAF.

### ANKLE2 mediates suppression of ΔB1 vaccinia virus DNA accumulation and plaque formation

**Fig 2:**
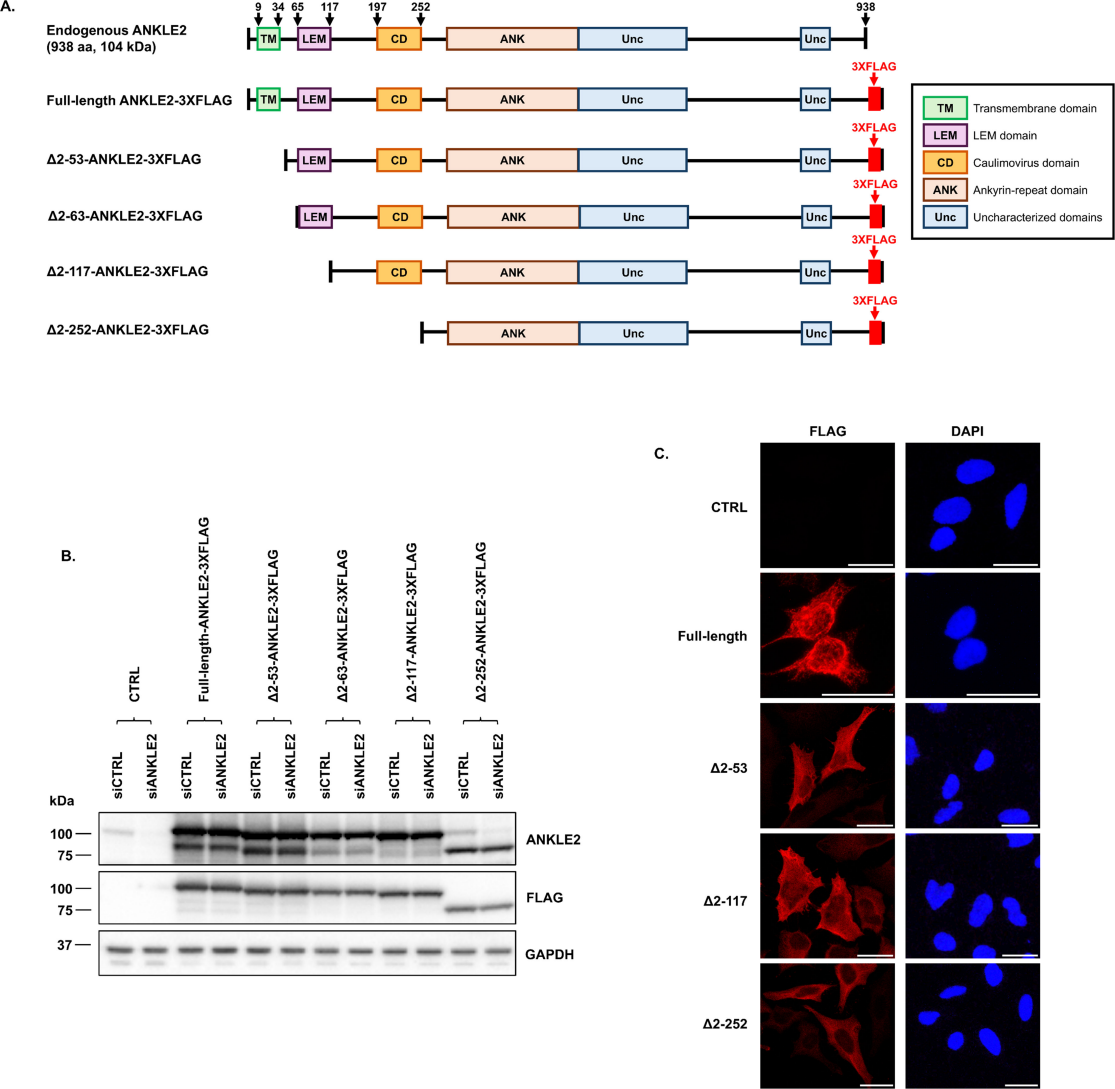
Characterization of expression and localization of endogenous and mutant ANKLE2 in siRNA-treated transduced HeLa cells. (**A**) Schematic representation of human full-length ANKLE2 and its N-terminal truncation mutants used in this study, showing annotated domains and amino acid positions indicated by black arrows. (**B**) HeLa cells were transduced with lentivirus expressing various siRNA-resistant ANKLE2 truncations, followed by transfection with siCTRL or siANKLE2. Cells were then treated with 1 µg/mL doxycycline for 3 days to induce protein expression and analyzed by immunoblotting. (**C**) Immunofluorescence analysis of HeLa cells transduced with empty vector (CTRL), full-length ANKLE2-3XFLAG, and N-terminal truncation mutants Δ2–53, Δ2–117, and Δ2–252. ANKLE2-transduced HeLa cells were stained with anti-FLAG antibody (red) and DAPI (blue). The representative images shown were taken using an inverted confocal microscope at ×60 magnification. Scale bars represent 25 µm.

We next sought to perform a structure/function analysis of ANKLE2 to characterize its posited antiviral capability. ANKLE2 is comprised of several conserved domains, including a N-terminal transmembrane domain (TM), a LEM domain known to bind BAF, a Caulimovirus-homology domain (CD), a central ankyrin-repeat (ANK) domain, and two uncharacterized regions (Unc) of unknown function, but predicted to be structured (33). To investigate the contribution of the TM, LEM, and CD domains of ANKLE2 in BAF dephosphorylation, we generated HeLa cell lines stably expressing siRNA-resistant versions of full-length ANKLE2 or one of four different N-terminal truncation mutants, including two deletions of the TM domain alone (Δ2–53 and Δ2–63), TM and LEM domains (Δ2–117), or TM through CD domains (Δ2–252) (Fig. 2A). Two distinct TM deletions were employed to allow for direct comparison with previous studies (35, 43, 44) and more rigorously assess the impact of TM domain loss during some studies discussed below. All mutants possess a C-terminal FLAG-epitope tag and were expressed using an identical doxycycline-inducible lentiviral expression system. A control HeLa cell line transduced with lentivirus encapsulating the empty (CTRL) vector was also used.

With these tools in hand, expression and localization of transduced ANKLE2 constructs were confirmed by immunoblotting and immunofluorescence assay (IFA) (Fig. 2B and C). IFA revealed that transduced full-length ANKLE2 was predominantly localized to membrane-associated regions (Fig. 2C), suggestive of association with the ER membrane and nucleus as previously reported (35, 36). All N-terminal truncation mutants exhibited modestly more diffuse staining (Fig. 2C), probably consistent with greater cytoplasmic distribution in the absence of the TM domain known to direct ANKLE2 to the ER (43). Next, to ensure that only transduced ANKLE2 contributed to the effects on vaccinia virus DNA replication and viral yield in subsequent experiments, siANKLE2 was used to specifically deplete endogenous ANKLE2. Immunoblotting confirmed siANKLE2 selectively depleted endogenous ANKLE2 levels in control cells, without altering transduced ANKLE2 protein expression as shown with either the ANKLE2 specific antibody or the FLAG antibody (Fig. 2B). A lower band on the ANKLE2 blot is occasionally detected using this antibody but absent on the FLAG blot, indicating it is either a non-specific signal or a degradation product from which the FLAG-epitope has been cleaved.

**Fig 3:**
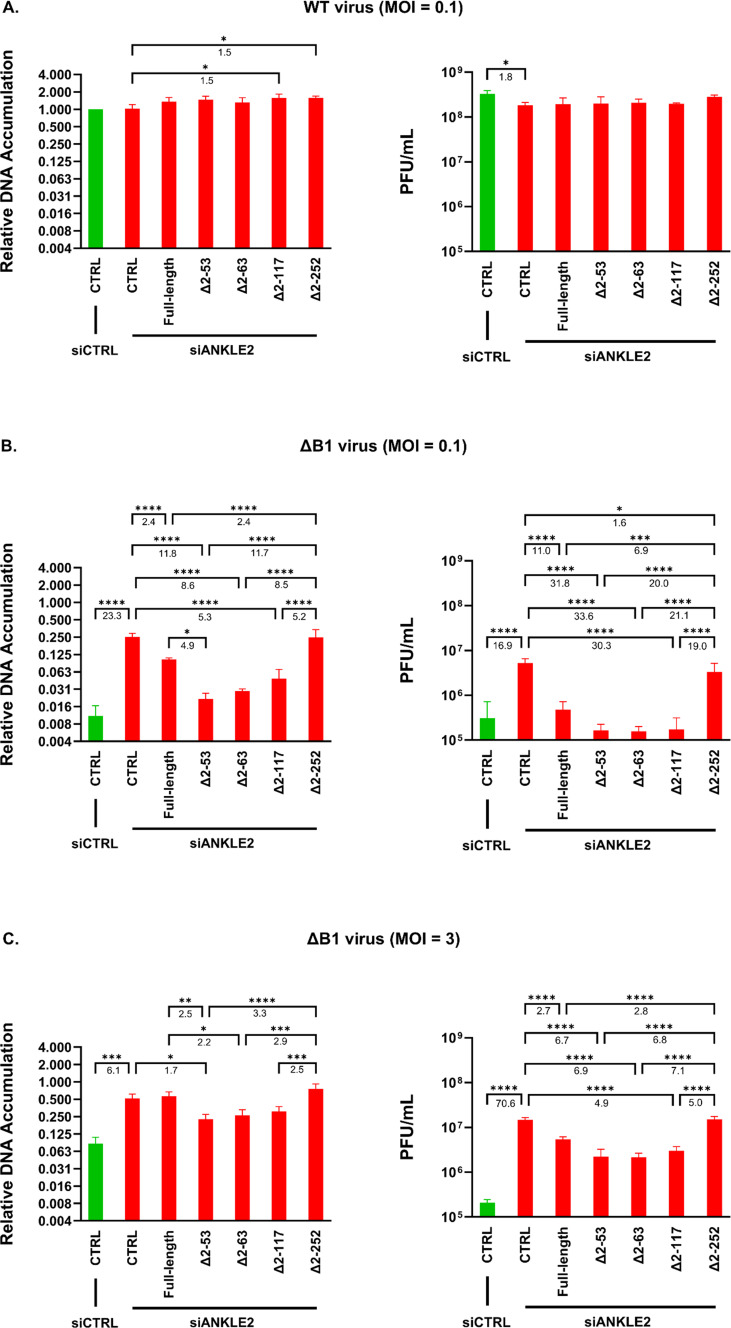
DNA accumulation and viral yield analysis of WT and ΔB1 vaccinia virus in ANKLE2-transduced HeLa cells following endogenous ANKLE2 depletion. DNA accumulation (left panels) and viral yield (right panels) were measured for (**A**) WT and (**B**) ΔB1 vaccinia virus at a low MOI of 0.1 at 48 hpi, as well as for (**C**) ΔB1 vaccinia virus at a higher MOI of 3 with 24 hpi. Data are from more than three independent experimental replicates. Numbers below the significance bars represent the average fold-change value. *, *P*  <  0.05; **, *P*  <  0.01; ***, *P*  <  0.001; ****, *P*  <  0.0001.

Next, following siANKLE2 transfection, transduced cells were infected with either WT or ΔB1 vaccinia virus at an MOI of 0.1 and processed at 48 hpi to determine DNA and viral yield. For the WT virus, a modest decrease (1.8-fold) in progeny production was observed following endogenous ANKLE2 depletion compared with control cells (Fig. 3A, right). Overexpression of full-length ANKLE2 or either TM domain-deleted mutants (Δ2–53 and Δ2–63) had no significant effect on WT DNA accumulation or viral yield (Fig. 3A), whereas cells expressing Δ2–117 or Δ2–252 ANKLE2 showed a minor increase (1.5-fold) in DNA accumulation relative to control cells (Fig. 3A, left). However, this increase did not translate into enhanced production of WT viral progeny (Fig. 3A, right).

An antiviral role for ANKLE2 was more clearly demonstrated against ΔB1 vaccinia virus. Specifically, after endogenous ANKLE2 depletion, ΔB1 DNA accumulation and viral yield were rescued by 23.3-fold and 16.9-fold, respectively, in control cells (Fig. 3B). Next, comparing ANKLE2-reconstituted cell lines treated with siANKLE2, full-length ANKLE2 expression significantly suppressed ΔB1 DNA accumulation by 2.4-fold (Fig. 3B, left), whereas progeny production was reduced by 11.0-fold (Fig. 3B, right) compared with the control cell. The ΔB1 DNA accumulation was even further suppressed in cells expressing Δ2–53, Δ2–63, and Δ2–117 ANKLE2 mutants, with reductions of 11.8-fold, 8.6-fold, and 5.3-fold, respectively, compared with control cells (Fig. 3B, left). This inhibition corresponded to 31.8-fold, 33.6-fold, and 30.3-fold decreases in viral yield, respectively (Fig. 3B, right). Meanwhile, cells expressing Δ2–252 ANKLE2 exhibited the smallest decrease (1.6-fold) in ΔB1 viral yield (Fig. 3B, right) of the truncation mutants, with no difference in DNA accumulation compared to control cells (Fig. 3B, left). Commenting on these results in more detail, it is interesting that deletion of the TM domain (Δ2–53) significantly enhanced suppression of ΔB1 DNA replication by 4.9-fold compared with reconstituted full-length ANKLE2 (Fig. 3B, left). A similar trend was observed with the other TM domain-deleted mutant (Δ2–63), which showed a 3.5-fold increase (*P* = 0.0852) in suppression (Fig. 3B, left) and suggests that the TM domain may limit the capacity of ANKLE2 to modulate antiviral activity.

Next, ΔB1 DNA replication and viral yield were measured after synchronous infection at a higher MOI (MOI = 3), revealing trends consistent with those observed at the lower MOI. Specifically, a rescue of both ΔB1 DNA replication (6.1-fold) and viral yield (70.6-fold) was found after endogenous ANKLE2 depletion in control cells (Fig. 3C). When comparing the various ANKLE2 mutants, although the magnitude of viral suppression by transduced ANKLE2 was less at a higher MOI than at the lower MOI, the TM domain-deleted ANKLE2 mutants (Δ2–53 and Δ2–63) showed the most robust inhibitory effect on ΔB1 DNA replication and viral yield. This was followed by the TM and LEM domain-deleted mutant (Δ2–117) and the full-length ANKLE2, respectively. Finally, a further deletion to remove the CD domain (Δ2–252) almost completely negated any impact this mutant had on ΔB1 DNA replication and viral yield, with levels of each reaching those closely matched by those of control cells at both low and high MOI infections (Fig. 3B and C).

**Fig 4:**
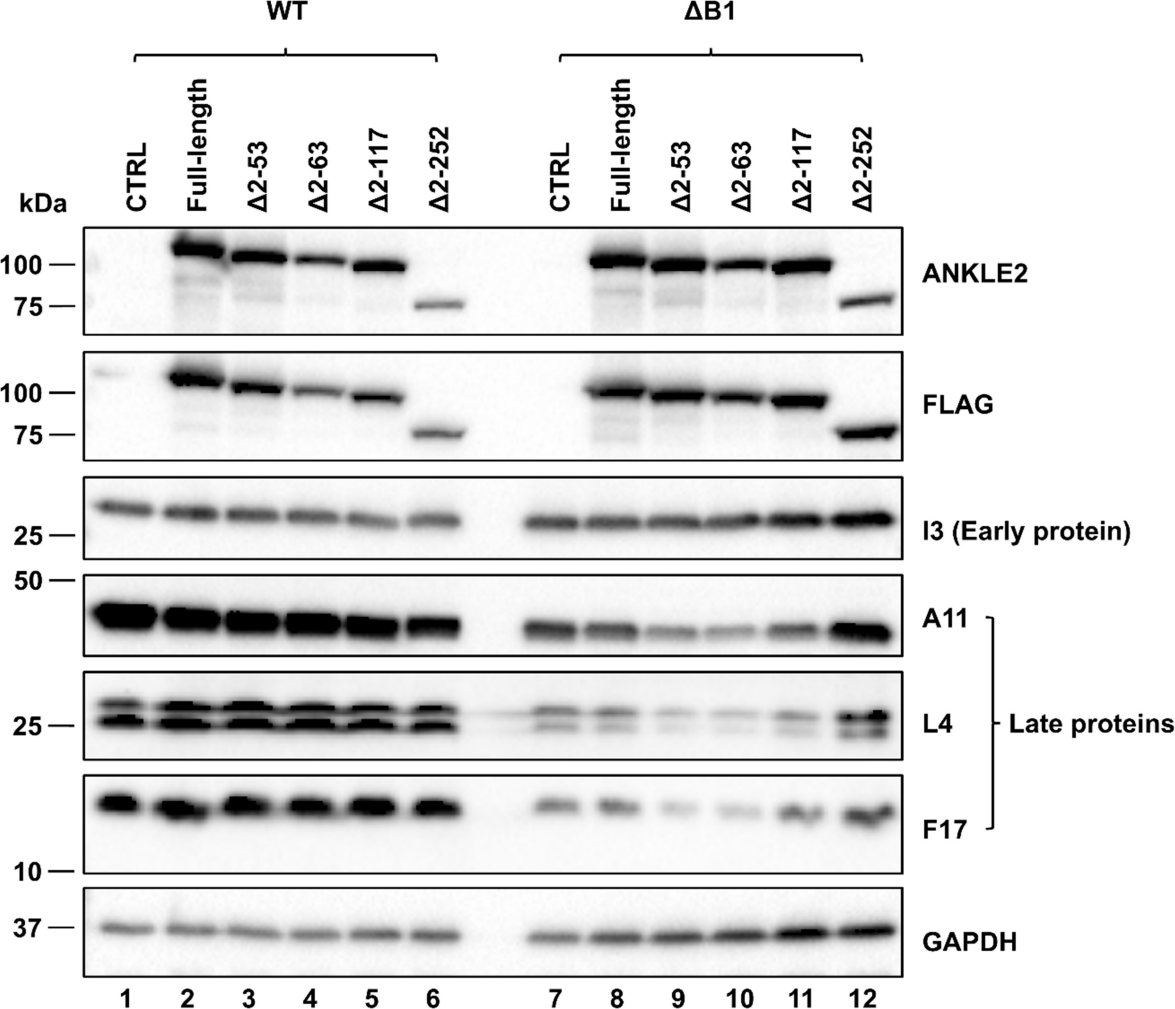
Abundance of viral proteins I3, A11, L4, and F17 during expression of ANKLE2 mutants. ANKLE2-transduced HeLa cells were transfected with siRNAs to deplete endogenous ANKLE2, followed by doxycycline inducement and infection with WT or ΔB1 vaccinia virus at an MOI of 3 for 24 hpi. Whole cell lysates were analyzed by immunoblot using antibodies specific to the proteins on the right. A representative immunoblot is shown with lane numbers shown below each column.

To further characterize the vaccinia virus life cycle in transduced cells, we assessed the expression of representative viral early and late proteins following infection (Fig. 4). Early protein expression, measured by viral I3 expression, was comparable across all transduced cell lines during both WT and ΔB1 vaccinia virus infections (Fig. 4). No differences were observed in the expression levels of late viral proteins A11, L4, and F17 during WT infection across all cell types either (Fig. 4, compare lanes 1–6). In contrast, during ΔB1 vaccinia virus infection, expression of late viral proteins was markedly decreased in cells expressing the TM domain-deleted mutants (Δ2–53 and Δ2–63) (Fig. 4, compare lanes 7 and 9–10). Additional deletion of the LEM domain (Δ2–117) resulted in a slight increase in late protein expression compared with the TM domain-deleted mutants (Fig. 4, compare lanes 9–10 and 11). Notably, deletion of the CD domain (Δ2–252) fully restored the late protein expression to levels comparable with those of control cells (Fig. 4, compare lanes 7 and 12). Together, these findings are consistent with the ability of ANKLE2 mutants to mediate suppression of ΔB1 vaccinia virus replication (Fig. 3B and C) in a manner independent of early gene expression and manifesting first at the stage of DNA replication and continuing into late protein abundance. Our data further indicate that the N-terminus of ANKLE2 contains elements that can both positively and negatively regulate the impact this protein has on vaccinia infection. The CD domain appears most important for activity, whereas the LEM domain is not strictly required but provides a significant contribution, and the TM domain acts as a potential negative regulator.

### Insights into ANKLE2 mechanism of action using immunofluorescence and subcellular fractionation

**Fig 5:**
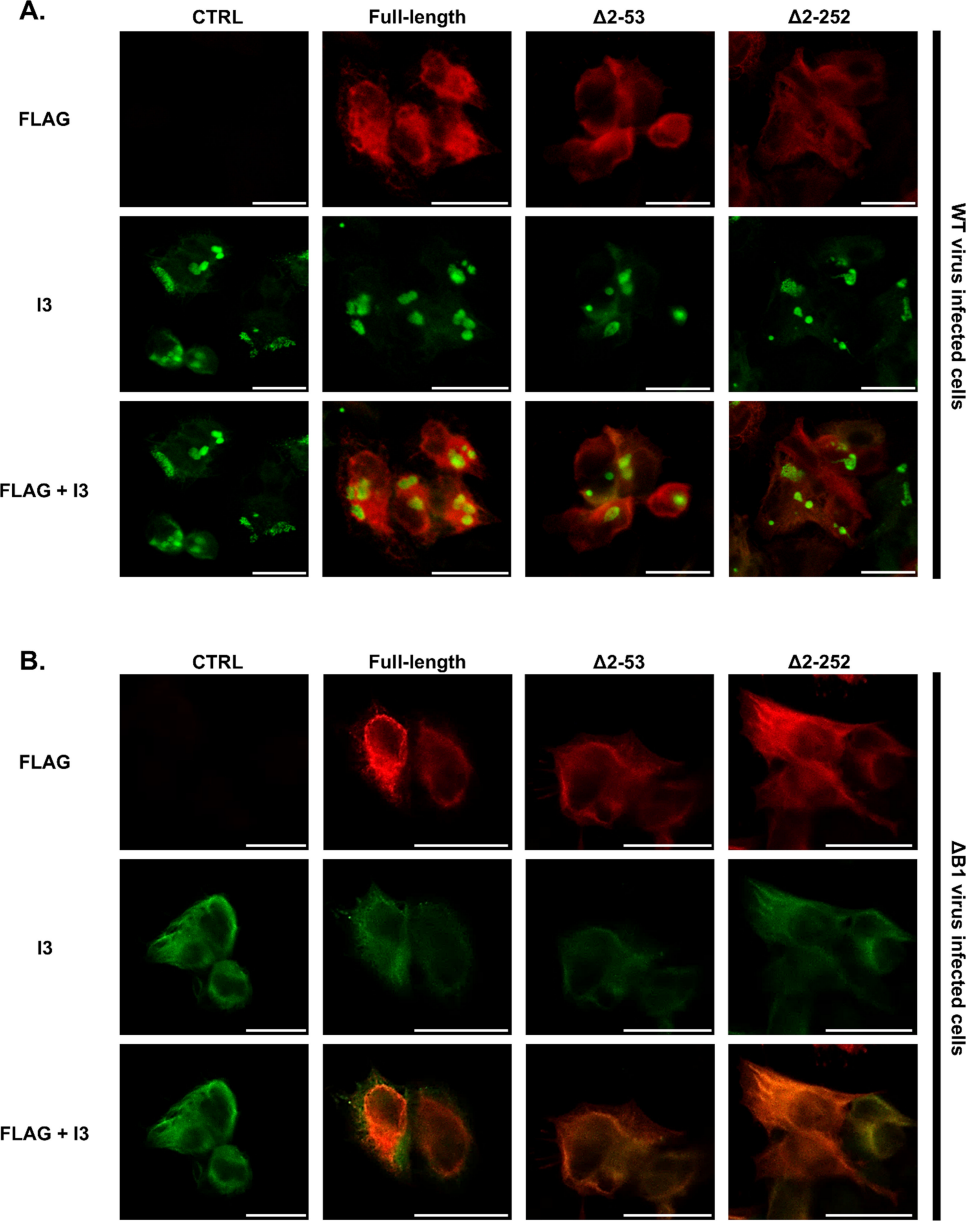
Immunofluorescence analysis of doxycycline-treated ANKLE2-transduced HeLa cells infected with (**A**) WT or (**B**) ΔB1 vaccinia virus at an MOI of 5 for 6 hours at 37°C prior to fixation. Viral DNA replication factories were detected by I3 staining (green) using anti-I3 antibody, whereas transduced ANKLE2 was detected using anti-FLAG primary antibody (red). The representative images shown were taken using an inverted confocal microscope at ×60 magnification. Scale bars represent 25 µm.

Previous studies have shown that orthoflaviviruses can trigger ANKLE2 relocalization to support their replication (39). To investigate whether transduced ANKLE2 proteins are redirected to vaccinia factories in a manner that contributes to viral suppression, we examined the relocalization of transduced ANKLE2 (full-length, Δ2–53, and Δ2–252 mutants) and the viral I3 by immunofluorescence (Fig. 5). As expected, at 6 hpi, I3-positive replication factories were observed in WT-infected cells (Fig. 5A), marking sites of viral factories, but no co-localization of ANKLE2 and I3 was observed. We also looked in ΔB1-infected cells, where I3 was diffusely distributed throughout the cytoplasm, as expected due to reduced DNA replication. Here, we again did not detect notable relocalization of transduced ANKLE2 (Fig. 5B). These data indicate that either direct localization to viral factories is unlikely to be needed for the antiviral capability of ANKLE2 or that ANKLE2 localization is somehow inhibited during WT infection.

**Fig 6:**
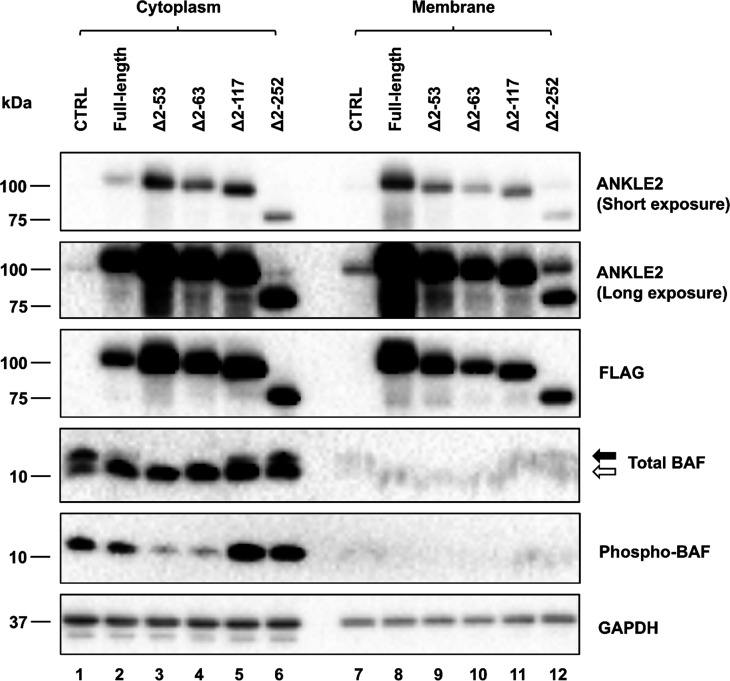
Protein immunoblotting of cytoplasmic BAF in doxycycline-treated ANKLE2-transduced HeLa cells following subcellular fractionation. Cytoplasmic and membrane fractions were analyzed by immunoblot using antibodies specific to the proteins on the right. When using an antibody recognizing total BAF, the closed arrow indicates the migration position of phosphorylated BAF, and the open arrow indicates the unphosphorylated BAF. A representative immunoblot is shown with lane numbers shown below each column.

To complement these immunofluorescence studies, we also performed subcellular fractionation followed by immunoblotting in the transduced cells. This approach also allows us to employ the phosphorylated BAF-specific antibody, which is not compatible with immunofluorescence. Using this approach, we found that all ANKLE2 mutants were detectable in both cytoplasmic and membrane fractions, albeit in different proportions. As expected, the transduced full-length ANKLE2 predominantly localized to the membrane-associated fractions (Fig. 6, compare lanes 2 and 8), whereas the N-terminal truncation mutants exhibited a greater distribution to the cytoplasm (Fig. 6, compare lanes 3–6 and lanes 9–12, respectively). When monitoring the phosphorylation state of BAF, we detected a decrease in phosphorylated cytoplasmic BAF in cells overexpressing full-length ANKLE2 (Fig. 6, compare lanes 1 and 2), and this effect was further enhanced in cells expressing the TM domain-deleted (Δ2–53 and Δ2–63) mutants of ANKLE2 (Fig. 6, lanes 3, 4). In contrast, deletion of the LEM domain (Δ2–117) resulted in a considerable increase in BAF phosphorylation levels (Fig. 6, lane 5, also indicated by the closed arrow in the total BAF blot). Finally, BAF phosphorylation in cells expressing the CD domain deletion (Δ2–252) (Fig. 6, lane 6) was indistinguishable from the CTRL cells. These data suggested that removing the TM domain results in more cytoplasmic redistribution of ANKLE2, perhaps facilitating even stronger BAF interactions and subsequent reduction of BAF phosphorylation, an effect mediated moderately by the LEM domain and mainly by the CD domain.

### ANKLE2 co-purification with BAF is regulated by its TM and LEM domain

**Fig 7:**
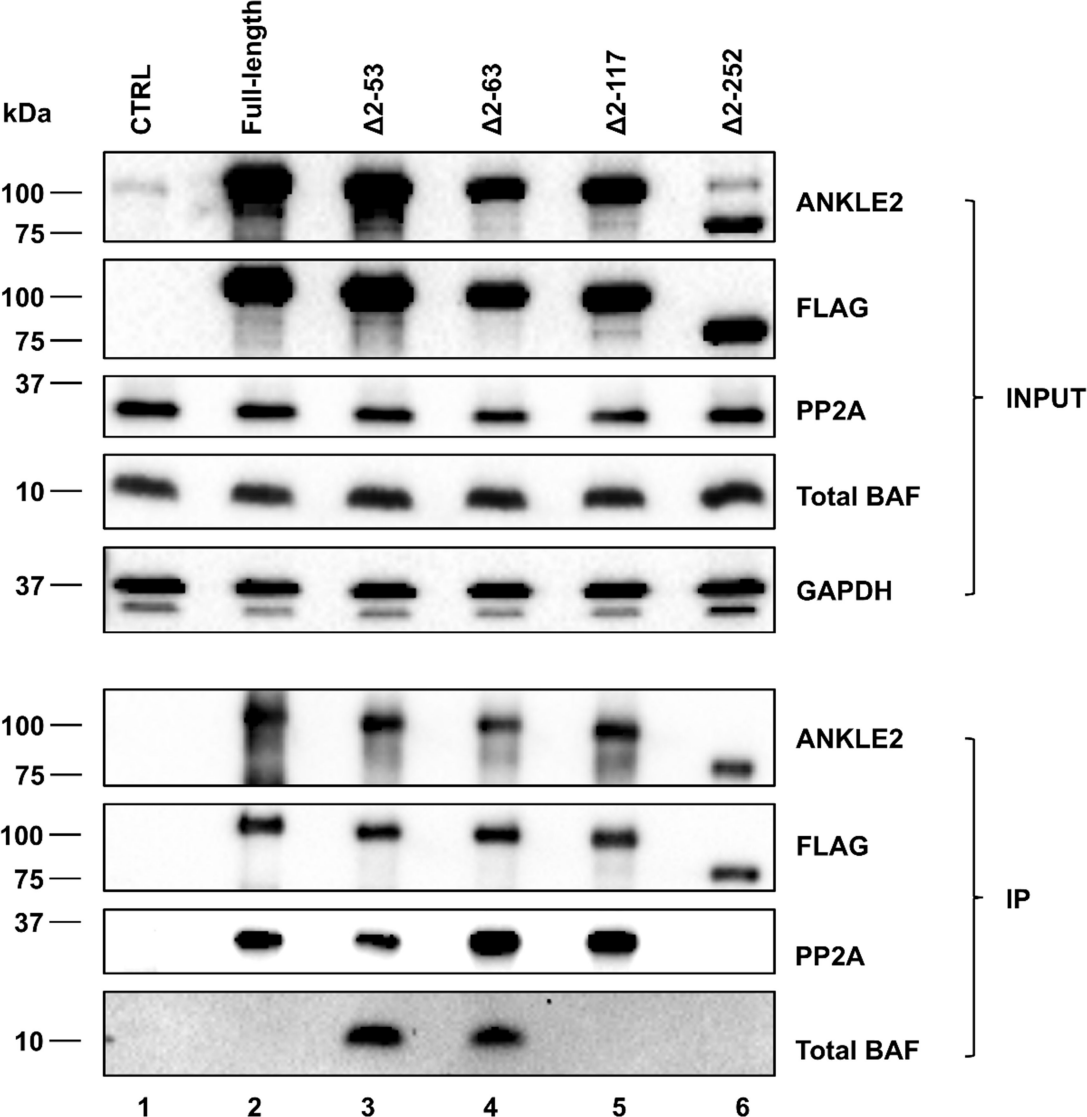
Immunoblotting of FLAG-tag immunoprecipitated lysates from doxycycline-treated ANKLE2-transduced HeLa cells. Confluent monolayers of cells in 10 cm^2^ dishes were harvested, pelleted, and lysed with a lysis buffer. Complexes of transduced ANKLE2 containing 3XFLAG tag were immunoprecipitated overnight at 4°C and eluted by incubation at 37°C for 24 h. Samples were then analyzed by immunoblotting using antibodies against ANKLE2, FLAG, PP2A, and total BAF proteins. INPUT lysates were included to confirm the similar levels of ANKLE2, PP2A, and BAF proteins in each lysate. A representative immunoblot is shown with lane numbers shown below each column.

ANKLE2 has been reported to physically interact with PP2A, resulting in PP2A-mediated dephosphorylation of BAF (35). Therefore, to further investigate the interaction between transduced ANKLE2, PP2A, and BAF, we performed co-immunoprecipitation (Co-IP) using FLAG antibody-conjugated Dynabeads, followed by immunoblotting. Immunoblots of parallel INPUT lysates were performed to verify the equivalent expression of ANKLE2, PP2A, and BAF proteins in each lysate (Fig. 7, INPUT). Immunoblotting for FLAG verified closely comparable levels of ANKLE2-FLAG in each precipitate, ensuring valid comparison among mutants (Fig. 7). Under these conditions, we were able to copurify PP2A and BAF from immunoprecipitates of cells expressing the TM domain-deleted (Δ2–53 and Δ2–63) mutants ANKLE2 (Fig. 7, lanes 3–4). In contrast, full-length ANKLE2 and the LEM domain-deleted mutant (Δ2–117) pulled down only PP2A (Fig. 7, lanes 2 and 5), whereas the CD domain-deleted mutant (Δ2–252) failed to copurify either PP2A or BAF (Fig. 7, lane 6). These findings suggest that removal of the TM domain, which anchors ANKLE2 to the endoplasmic reticulum and nuclear envelope, also enhances BAF co-purification. Compared with the ∆TM mutants, further truncation of ANKLE2 correlates with the loss of BAF coprecipitation. As the LEM domain is absent in both Δ2–117 and Δ2–252 mutants, these data are consistent with prior evidence that the LEM directly binds BAF, whereas the CD domain interacts with PP2A, which subsequently modulates BAF (35). Together, this study indicates for the first time that ANKLE2-BAF interaction contributes to ANKLE2 antiviral activity.

## DISCUSSION

**Fig 8:**
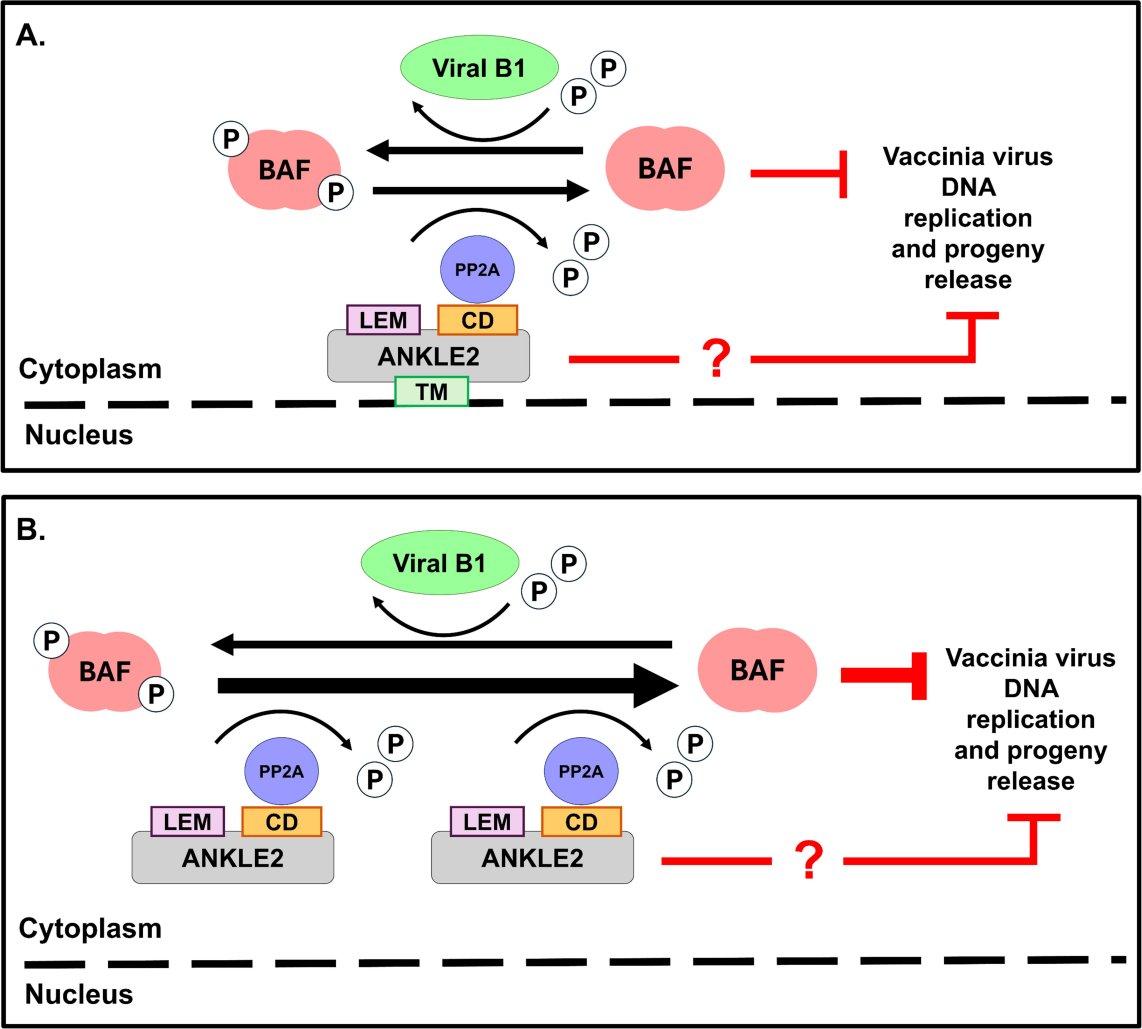
Working model of ANKLE2 antiviral activity against vaccinia virus. The LEM domain mediates direct interaction with BAF, whereas the CD domain interacts with PP2A, which subsequently dephosphorylates BAF. (**A**) The TM domain anchors ANKLE2 to the endoplasmic reticulum (ER) and nuclear envelope, thereby limiting these interactions. (**B**) Deletion of the TM domain results in cytoplasmic relocalization of ANKLE2, enhancing its interaction with BAF, which promotes BAF dephosphorylation and suppresses viral DNA replication and progeny release. Thick T-shaped red lines indicate stronger inhibition, and thin red T-shaped lines indicate normal inhibition. Thick black arrows indicate stronger reaction events, and thin black arrows indicate normal reaction events.

ANKLE2 plays critical, evolutionarily conserved roles in cell division and development, as well as serving an intriguing proviral function during replication of Zika virus (37, 39, 45). During cell division, ANKLE2 serves as a scaffold to regulate dephosphorylation of BAF via modulation by PP2A (35). Herein, our study demonstrates a novel antiviral role of ANKLE2 in suppressing vaccinia virus DNA replication and progeny release through the regulation of BAF phosphorylation (Fig. 8). We provide clear evidence that depletion of endogenous ANKLE2 disrupts BAF dephosphorylation, leading to an increase in phosphorylated BAF. This modification is already known to be less effective in restricting viral DNA replication (3, 8) and consequently promotes ΔB1 vaccinia virus replication and progeny production.

We investigated the molecular mechanism through which ANKLE2 suppressed vaccinia replication by reconstituting ANKLE2 expression in siRNA-treated cells with full-length ANKLE2 or a panel of truncation mutants. Overexpression of full-length ANKLE2 resulted in reproducible suppression of ΔB1 viral DNA replication and progeny release, indicating that full-length ANKLE2 contributes to antiviral defense. Interestingly, though, removal of the N-terminal TM domain markedly enhanced the antiviral activity of ANKLE2, significantly increasing suppression of viral DNA replication and progeny production compared with the full-length ANKLE2. The increased suppression upon loss of the TM region was unexpected but clearly observed in two modestly different truncation mutants chosen based on their prior use in other ANKLE2 studies to confirm this property of the ΔTM mutant (35, 43, 44). This increased antiviral effect upon TM removal corresponded with a stronger ability to suppress BAF phosphorylation, consistent with the model that ANKLE2 is activating the antipoxviral activity of BAF, known to be governed by phosphorylation (8). Mechanistically, the TM domain deletion is known to cause a relocalization of ANKLE2 from the ER membrane and nuclear envelope to the cytoplasm (43). Consistent with this, our subcellular fractionation and immunofluorescence analyses indicate that loss of the TM domain results in reduced membrane association and increased cytoplasmic localization of ANKLE2. We posit that this cytoplasmic redistribution likely facilitates more efficient interaction with cytoplasmic BAF, which was enriched in co-immunoprecipitation assays with TM domain-deleted ANKLE2 mutants. Intriguingly, the TM domain of ANKLE2 appears absent from LEM4 homologs in zebrafish, flies, and worms, as well as from a human splice variant of ANKLE2 reported in some tissues (33, 43). Future studies will be needed to determine whether these homologs of ANKLE2 exhibit the antiviral properties we have described here.

Previous study of ANKLE2 function during mitosis demonstrated that a Lem4/ANKLE2 construct comprised of amino acids 59–938 (thus missing the TM domain) strongly inhibits BAF phosphorylation, whereas the removal of the LEM domain (aa 114–938) reduces this inhibitory effect, and further deletion including the CD and ANK domains (aa 442–938) completely abolishes it (35). In addition, the LEM domain is reported not to be required for ANKLE2 to bind PP2A, suggesting that this interaction remains intact even when the LEM domain is removed (35). Consistent with these findings, our data showed that deletion of the LEM domain reduces its ability to decrease BAF phosphorylation, whereas its interaction with PP2A remains intact, indicating that binding occurs independently of the LEM domain. This preserved binding may explain why deletion of the LEM domain incompletely impairs suppression of BAF phosphorylation and still allows modest antiviral activity against vaccinia virus DNA replication and progeny production. Meanwhile, deletion of the CD domain disrupted ANKLE2’s binding to PP2A. Without this critical interaction, ANKLE2 can no longer support PP2A-mediated dephosphorylation of BAF, resulting in a total loss of antiviral activity. Additional intriguing alternative explanations for the antiviral activity of the LEM deletion mutant are that ANKLE2 may be able to weakly bind BAF via a LEM-independent mechanism or that ANKLE2 can act through other unknown pathways.

Analysis of viral protein expression further supports our model that ANKLE2 restricts ΔB1 vaccinia virus primarily at the viral DNA replication stage. Early protein expression was comparable across all transduced lines in both WT and ΔB1 infections, suggesting that ANKLE2 does not impact the early stage of the vaccinia viral life cycle. However, late viral proteins (A11, L4, and F17) of ΔB1, but not WT, vaccinia virus were noticeably reduced in cells expressing TM domain-deleted mutants, partially relieved by deletion of the LEM domain, and fully ablated upon deletion of the CD domain. The selective modulation of ΔB1 vaccinia virus replication by ANKLE2 suggests that its antiviral effect occurs through regulation of BAF dephosphorylation, as previous findings have shown that BAF potently inhibits vaccinia virus replication unless its DNA-binding activity is neutralized by viral B1-mediated phosphorylation (4, 8).

In sum, our study uncovers a novel antiviral role of ANKLE2 in suppressing vaccinia virus DNA replication and progeny release by regulating the phosphorylation status of BAF (Fig. 8). This activity is shaped by ANKLE2’s localization and domain composition, with the TM domain modulating antiviral potency through altered interaction with BAF. The LEM and CD domains are important to three properties of ANKLE2: poxvirus repression, BAF hypophosphorylation, and ANKLE2-BAF copurification, indicating that these three events are mechanistically linked. However, although our findings support a mechanism involving BAF phosphorylation, other molecular pathways involving the C-terminal domains of ANKLE2 and possibly BAF-independent antiviral actions of ANKLE2 remain to be elucidated. Together, these insights highlight ANKLE2 as a host factor in poxvirus restriction and a potential target for antiviral intervention.

## MATERIALS AND METHODS

### Cell culture

Human kidney 293T cells were purchased from ATCC. Human cervix epithelial adenocarcinoma HeLa cells were purchased and obtained directly from the ATCC. African green monkey kidney CV1 cells were obtained from Invitrogen Life Technologies. These cell lines were maintained in high-glucose Dulbecco’s modified Eagle’s medium (DMEM/high glucose; Cytiva HyClone) supplemented with 10% fetal bovine serum (FBS; Atlanta Biologicals) and penicillin-streptomycin and incubated at 37°C in a 5% CO_2_ atmosphere.

### Plasmid construction

The codon-optimized full-length ANKLE2 open reading frame in the pcDNA3.1 vector was purchased from ThermoFisher. The full-length ANKLE2-3XFLAG ORF was digested with NheI and BamHI restriction enzymes and ligated into the pCW57-PURO-MCS1-2A-MCS2 vector to generate the pCW57-PURO-ANKLE2-3XFLAG plasmid. pCW57-MCS1-2A-MCS2 was a gift from Adam Karpf (Addgene plasmid # 71782). Each ANKLE2-3XFLAG N-terminal truncation mutant, including Δ2-53-ANKLE2-3XFLAG, Δ2-63-ANKLE2-3XFLAG, Δ2-117-ANKLE2-3XFLAG, and Δ2-252-ANKLE2-3XFLAG, was generated from the pCW57-PURO-ANKLE2-3XFLAG plasmid using the Q5 Site-Directed Mutagenesis Kit (New England Biolabs) with specific forward primers Δ2–53_Fwd (5′-GCCGCTGCTGCTCCAGCT-3′), Δ2–63_Fwd (5′-ACAATGGATGCCCTGCTG-3′), Δ2–117_Fwd (5′-AGCAGCTTCTACCACCATG-3′), or Δ2–252_Fwd (5′-ATCTGCGACTACTTCCCATCTC-3′), together with a shared reverse primer, pCW57_ANKLE2_del2-53/63/117/252_Rev (5′-CATGGTGGCGCTAGCCAA-3′). Plasmids were confirmed by DNA sequencing.

### Lentivirus production

The lentiviruses encapsulating pCW57-PURO-MCS1-2A-MCS2 (CTRL; control), pCW57-PURO-ANKLE2-3XFLAG, pCW57-PURO-Δ2-53-ANKLE2-3XFLAG, pCW57-PURO-Δ2-63-ANKLE2-3XFLAG, pCW57-PURO-Δ2-117-ANKLE2-3XFLAG, and pCW57-PURO-Δ2-252-ANKLE2-3XFLAG were produced in 293T cells. Cells were transfected with the individual plasmid along with pVSVG and psPAX2 helper plasmids using Lipofectamine 2000 (Thermo Fisher Scientific) (7). Fresh media supplemented with 5 mM sodium butyrate (EMD Millipore Corp.) and 1X GlutaMAX (Gibco) was added to cells 16 h post-transfection. At 24 h post-transfection, fresh media supplemented with 10 mM HEPES (Fisher Scientific) and 1X GlutaMAX (Gibco) were added to the cells. Virus in the supernatant was collected at 48 h post-transfection and filtered through a 0.45 µm syringe. Polybrene (Fisher Scientific) was added to lentivirus at 10 µg/mL and used immediately to generate the stably transduced cell lines.

### Generation of stably transduced cell lines

To produce stably transduced cell lines, HeLa cells were seeded at 25% confluence in 35 mm dishes. After 24 h, the culture medium was replaced with 0.8 mL of each lentiviral supernatant and incubated for 16 h. The cell supernatant was then removed and replaced with fresh growth medium, followed by an additional 24 h incubation. To select for stable lentiviral integration, the cells were passaged in a medium containing 1 µg/mL puromycin. After selection, the cells were induced for 3 days with 1 µg/mL doxycycline to induce protein expression. Protein expression was confirmed by immunoblotting using a mouse anti-FLAG M2 (Sigma) antibody.

### siRNA depletion

Small interfering RNAs (siRNAs) against ANKLE2 (siANKLE2) and nontargeting siRNAs (siCTRL) have been previously validated (35) and were ordered from Dharmacon. The siRNA sequences were as follows: siANKLE2 (5′-GGUCAUAUGUUUAUUGCUAUU-3′), siBAF (5′-GGCCUAUGUUGUCCUUGGCUU-3′), and siCTRL (5′-CAGUCGCGUUUGCGACUGGUU-3′). Cells were transfected with 10 nM siRNA using Lipofectamine RNAiMAX (Life Technologies) according to the manufacturer’s protocol. Protein depletion was measured by immunoblot analysis at 3 days post-transfection, and the cells were also used for other downstream assays at 3 days post-transfection.

### Viruses and viral infections

Wild-type (WT) and B1-deleted virus (ΔB1) WR strain vaccinia viruses purified as described (31) were used in this study. For vaccinia viral DNA accumulation and viral titer determination, confluent monolayers of cells were infected with WT or ΔB1 virus at a multiplicity of infection (MOI) of 0.1 or 3 at 37°C for 48 or 24 h, respectively. Cells were harvested and divided into two equal aliquots for viral DNA accumulation and viral yield assays. For viral yield assays, following cell harvest, cells were pelleted and resuspended in 200 µL of 10 mM Tris (pH 9). Virus samples were freeze-thawed three times and titrated on complementing CV1B1 cells produced as previously described (31). Cells were fixed and stained at 72 h post-infection. For immunofluorescence assays, confluent monolayers of cells were infected with WT or ΔB1 virus at an MOI of 5 at 37°C for 6 h.

### DNA purification and qPCR

Vaccinia viral DNA was extracted using the GeneJET Whole Blood Genomic DNA Purification Mini Kit (Thermo Scientific) according to the manufacturer’s protocol. Quantitative PCR (qPCR) was performed using iTaq Universal SYBR Green Supermix (Bio-Rad) with specific primers targeting the vaccinia virus hemagglutinin (HA) gene: HA_Fwd (5′-CATCATCTGGAATTGTCACTACTAAA-3′) and HA_Rev (5′-ACGGCCGACAATTATAATTAATGC-3′) primers as previously described (42). Serial dilutions of vaccinia DNA were included in each qPCR run to generate a standard curve and determine the PCR efficiency of the primer set. Each qPCR reaction was performed using approximately 10 ng of DNA and 1 µM of each primer.

### Subcellular fractionation assay

Cells were fractionated into soluble cytoplasmic, membrane, soluble nuclear, and chromatin-bound nuclear fractions using a subcellular protein fractionation kit for cultured cells (Thermo Scientific) according to the manufacturer’s instructions, with the addition of phosphatase inhibitors. GAPDH (glyceraldehyde-3-phosphate dehydrogenase) was used as a predominant cytosolic and minor membrane-associated protein control. Samples were evaluated by immunoblotting.

### Protein co-immunoprecipitations

To determine the protein interactome of ANKLE2, confluent monolayers of cells in 10 cm^2^ dishes were harvested in cold Dulbecco’s Phosphate Buffered Saline (DPBS; Cytiva HyClone) and pelleted. Cells were lysed with a lysis buffer containing 50 mM Tris-HCl (pH 7.4), 150 mM NaCl, and 1% Triton X-100, and then supplemented with protease and phosphatase inhibitors and nuclease (Pierce Universal Nuclease). Lysates were clarified following centrifugation, and ANKLE2-3XFLAG complexes were immunoprecipitated using Dynabead Protein G magnetic beads conjugated to mouse anti-FLAG antibodies (Sigma, F1804). Complexes were eluted in SDS sample buffer with protease and phosphatase inhibitors by incubating at 37°C for 24 h. Samples were evaluated by immunoblotting.

### Immunoblotting

To assess protein expression, cells were harvested, pelleted, and suspended in an SDS sample buffer supplemented with protease and phosphatase inhibitors and nuclease (Pierce Universal Nuclease). Whole-cell lysates were resolved on 12% or 18% SDS-PAGE gels and transferred to polyvinylidene difluoride membrane (Bio-Rad). Equal fractions of total cells were loaded for each condition without normalization to total protein concentration. GAPDH was used as a loading control to confirm that comparable numbers of cells were analyzed and that ANKLE2 depletion did not alter overall cell number. The primary antibodies used are as follows: rabbit anti-ANKLE2 (1:1,000; Novus Biologicals; NBP2-15396), mouse anti-FLAG (1:3,000; Sigma, F1804), rabbit anti-FLAG (1:1,000; Thermo Fisher Scientific, 740001), rabbit anti-GAPDH (1:1,000; Santa Cruz Biotechnology; sc-25778), rabbit anti-Total BAF (1:500; custom), rabbit anti-Phosphorylated BAF (1:100; custom), rabbit anti-L4 (1:2,000; custom), rabbit anti-A11 (1:2,000; custom), rabbit anti-I4 (1:2,000; custom), rabbit anti-F17/F18 (1:6,000; custom), and mouse anti-PP2A (1:1,000, Sigma; 05-421).

### Immunofluorescence assays

Cells were fixed in 4% paraformaldehyde in PBS and permeabilized with 0.2% Triton X-100 in PBS for 5 min at room temperature. The primary antibodies used were mouse anti-FLAG (1:400; Sigma, F1804) and rabbit anti-I3 (1:400; custom), followed by Alexa Fluor 594- and Alexa Fluor 488-conjugated secondary antibodies against FLAG and I3, respectively. Nuclei were counterstained with DAPI (4′,6-diamidino-2-phenylindole). Fluorescence images were acquired on an inverted confocal microscope (Nikon A1R-Ti2).

### Statistics

All experiments were performed in at least biological triplicate, and the graphed data represent the mean values of all experimental replicates. Error bars indicate one standard deviation (SD) from the mean. Statistical significance was assessed using one-way or two-way analysis of variance (ANOVA) or multiple *t*-tests, as appropriate, using GraphPad Prism version 10.5.0 for Windows.

## Data Availability

All unique reagents generated in this study are available from the lead contact. All other data supporting the findings of this study are contained within the article.
